# Case report: A rare case of malignant solitary fibrous tumor within the joint cavity with review of the literature

**DOI:** 10.3389/fonc.2024.1463362

**Published:** 2024-11-29

**Authors:** Xiao-Jie Wang, Jia-Ping Zhou, Yao Pan, Ri-Sheng Yu

**Affiliations:** Department of Radiology, Second Affiliated Hospital, Zhejiang University School of Medicine, Hangzhou, China

**Keywords:** solitary fibrous tumors, joint, computed tomography, magnetic resonance imaging, positron emission tomography-computed tomography

## Abstract

Solitary fibrous tumors (SFTs) are classified as fibroblastic/myofibroblastic tumors that originate from CD34-positive dendritic cells and usually occur in the pleura. In this paper, we describe a case of SFT within the joint cavity of the left knee. A 60-year-old man was admitted to hospital due to swelling in the left knee for the past 8 months without relevant trauma history. X-ray, computed tomography (CT), magnetic resonance imaging (MRI), and positron emission tomography–computed tomography (PET-CT) presented a large, ill-circumscribed, hypervascular, and highly enhanced mass with eccentric calcification and peripheral, intra-lesional vessels. Subsequently, the patient underwent surgical resection. Postoperative pathology confirmed the neoplastic cells to be positive for CD34, Bcl-2, and SATA6, therefore was finally diagnosed as malignant SFT. The patient developed bone metastases within 1 year after surgery. SFT in the joint cavity is rare, and it is difficult to make a preoperative diagnosis.

## Introduction

Klemperer and Rabin reported the first case of a fibrous mesothelioma located in the pleura and showed a benign clinical course (1). The understanding of solitary fibrous tumors (SFTs) has evolved over time. According to the recent World Health Organization (WHO) classification of soft tissue tumors, SFTs are classified as fibroblastic/myofibroblastic tumors that originate from CD34-positive dendritic cells, the biological behavior of which is further subdivided into intermediate/locally aggressive (SFT-benign), intermediate/rarely metastasizing (SFT-NOS, including fat-forming SFT and giant cell-rich SFT), and malignant (SFT-malignant) (2). Hemangiopericytoma was once used as a synonym for SFT occurring extra-pleura. Subsequently, giant cell angiofibroma and hemangiopericytoma were removed from the WHO classification of soft tissue tumors in 2013, with the former being classified as SFT outside the pleura, which was described as one morphologic variant, while the latter was recommended to be disregarded as a lot of soft tissue tumors could display a hemangiopericytomatous vasculature (3, 4). In addition, the *NAB2*–*STAT6* gene fusion was identified in SFTs in 2013 (5).

SFTs are relatively rarely seen in clinical practice, but occur in a wide variety of body sites (6–9). With regard to the imaging features of SFTs, there are no characteristic findings. Computed tomography (CT), magnetic resonance imaging (MRI), echocardiography, and positron emission tomography–computed tomography (PET-CT) imaging findings have been reported mainly in case series (10). Radiographs, echocardiography, and CT alone generally yield non-specific findings for SFTs, and the density of SFTs can be uniform or uneven. At ultrasound, SFTs are typically hypoechoic, but are occasionally heterogeneous. Calcification is rare, but could exist and can be observed using CT or by detecting the presence of bright echo-dense areas on echocardiography. Compared with those of CT, the imaging findings of MRI are more conducive to helping radiologists in analysis and assessment due to their high resolution of soft tissue and multi-parameter imaging. MRI can reflect the histological characteristics of SFTs. Low-signal-intensity foci on T1- and T2-weighted images, representing the collagen content, are a frequent feature of SFTs. As highly vascular tumors, SFTs are avidly enhanced on both CT and MRI. Whole-body imaging with PET-CT can be performed to determine multiple tumor sites and to detect metastatic lesions in the case of malignant SFTs. An increased uptake of fluorodeoxyglucose F18 (F-18 FDG), which corresponds to glucose metabolism, could indicate an aggressive or a malignant type of SFT (10, 11).

In this paper, we present a case of a malignant SFT within the joint cavity of the left knee, a rare site of occurrence, and the morphology of the mass led to this case being rare. We mainly focused on its radiological features, followed by a review of the literature. It is hoped that the study findings will be beneficial to radiologists in clinical work.

## Case presentation

**Figure 1:**
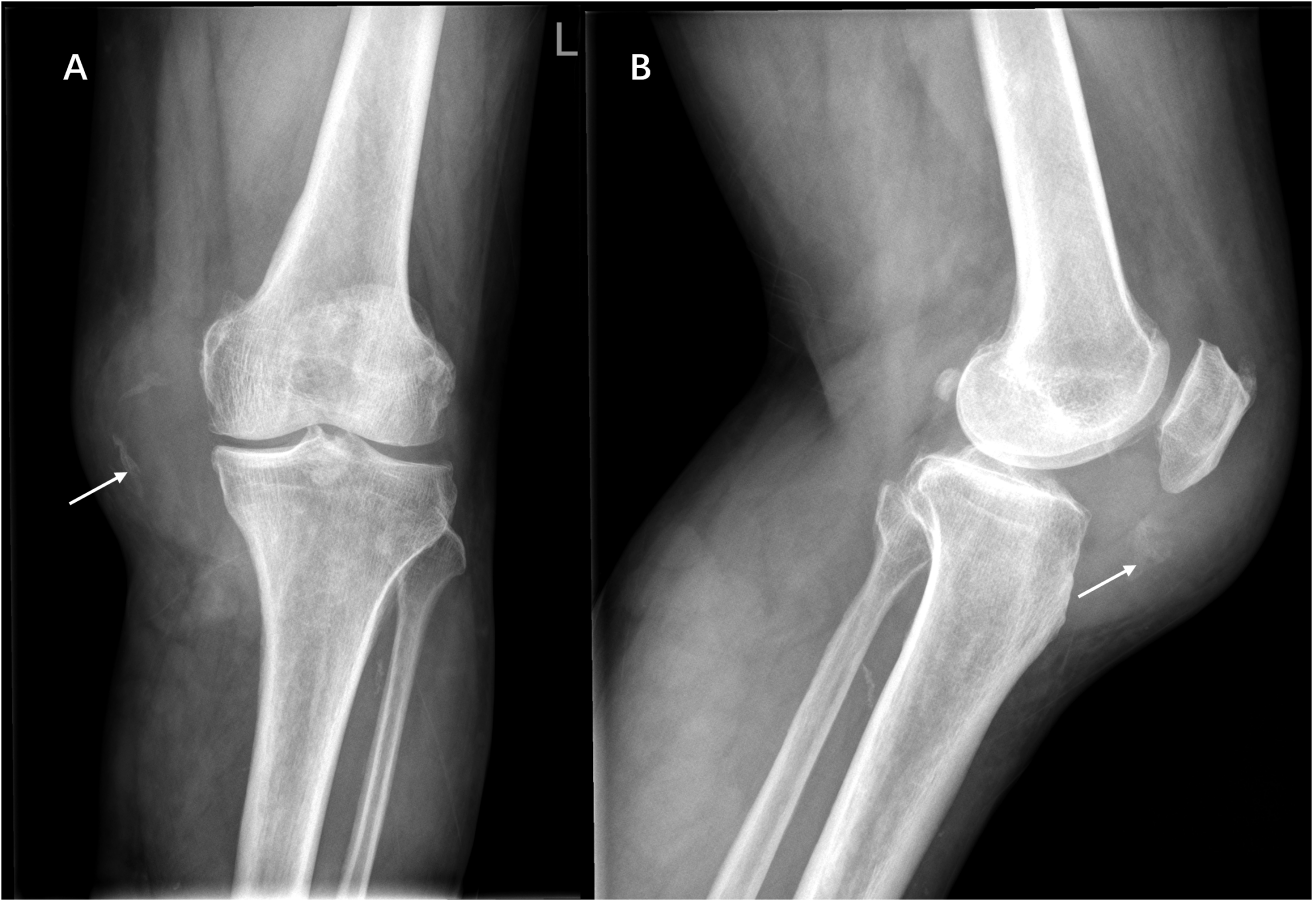
Anteroposterior **(A)** and lateral **(B)** radiographs showing marked swelling of the anteromedial soft tissue of the left knee with ill-defined borders and scattered calcifications within it (*white arrow*), without bone destruction.

**Figure 2:**
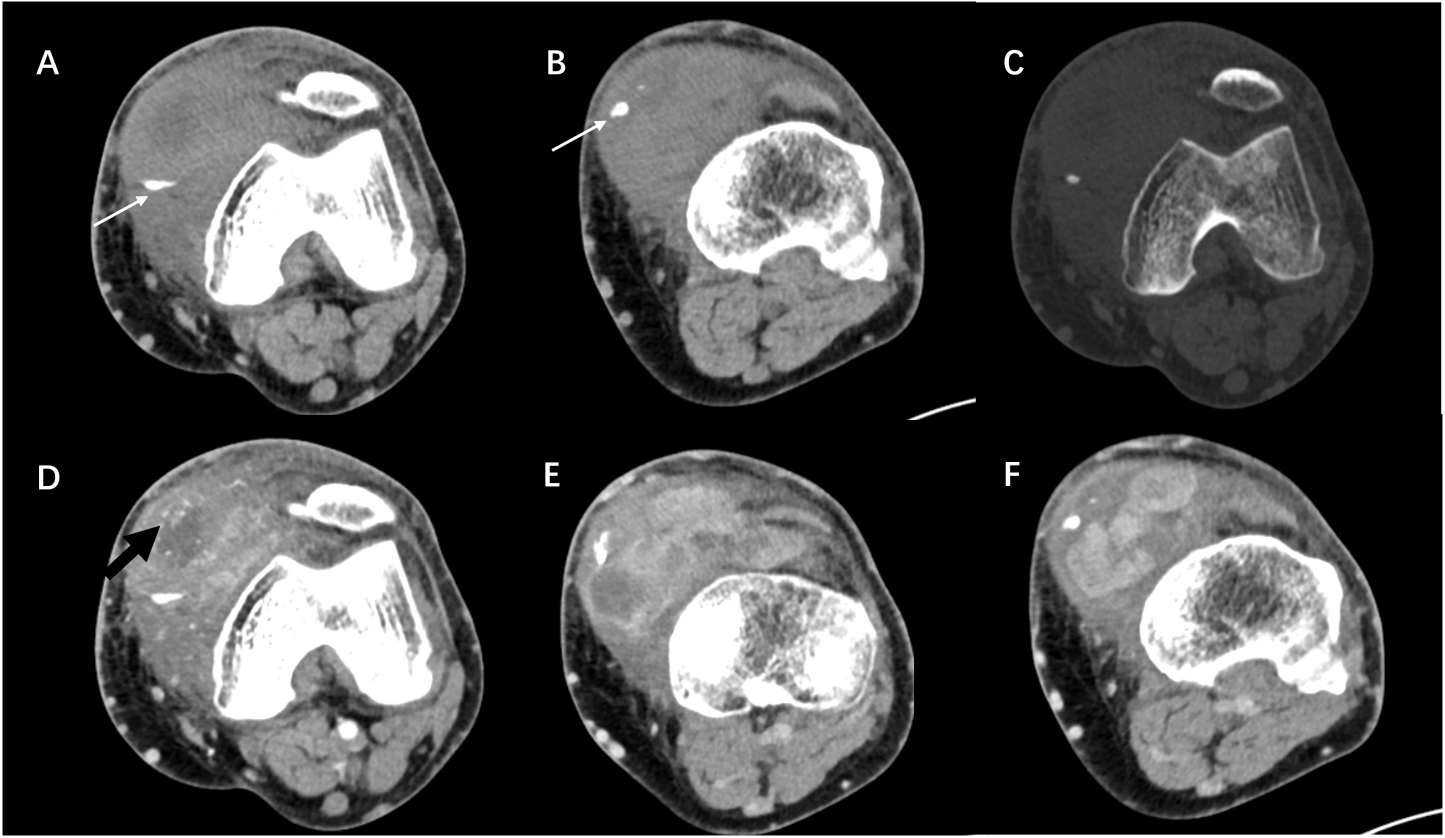
**(A–C)** Axial CT images showing a soft tissue mass in the anteromedial side of the left knee, which was irregular in shape and ill-defined in boundary, without bone destruction. The calcification was mainly located at the edge of the lesion (*white arrow*). **(D–F)** Contrast-enhanced scan showing the markedly heterogeneous enhancement of the mass. Nodular enhancement and enhanced vessels (*black arrow*) could be seen within the mass.

**Figure 3:**
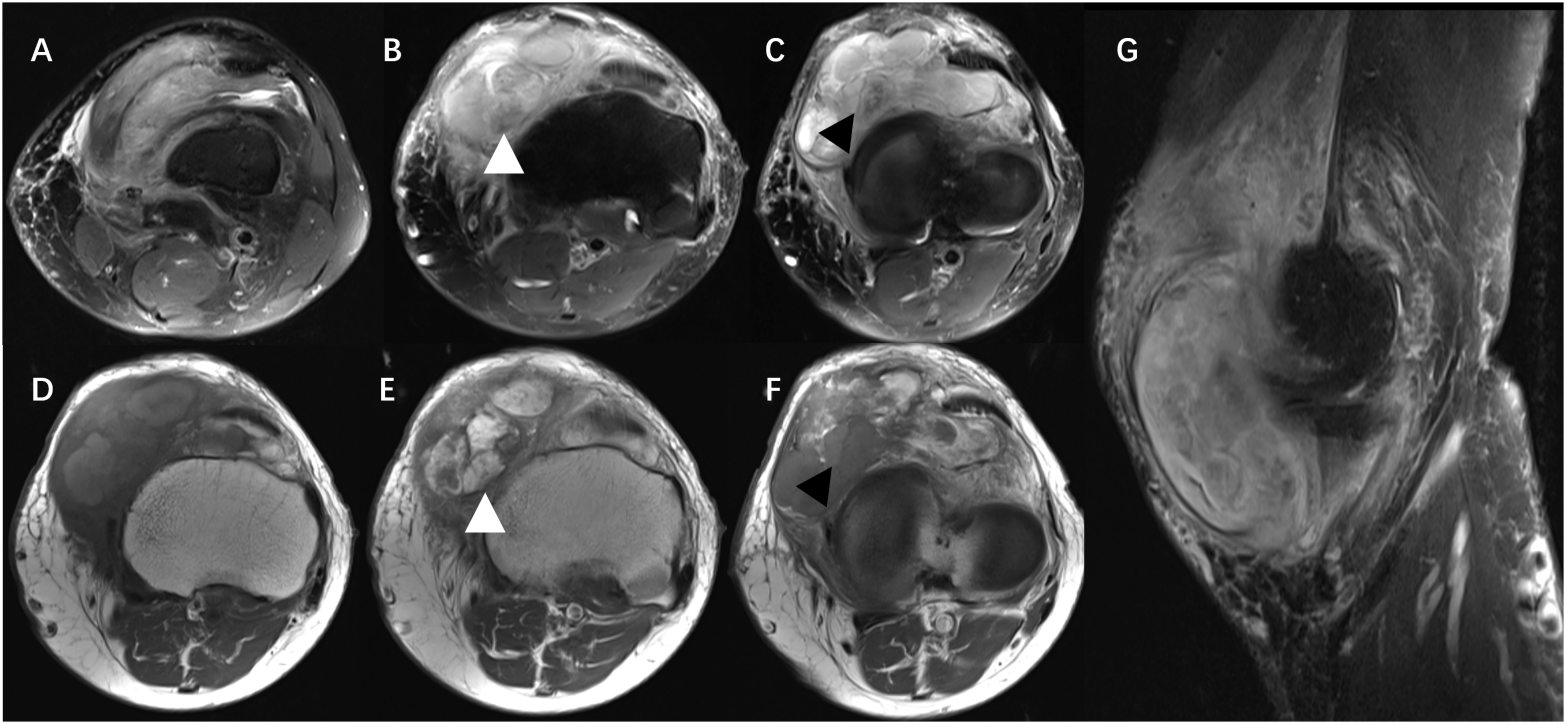
MRI showing that the mass was mainly located in the joint cavity of the left knee, with an irregular shape, unclear boundary, and mixed signals. On T2-weighted images (T2WI), the lesion was hyperintense, with the adjacent medial collateral ligament of the left knee swollen and partly invaded by the mass **(A–C)**. On T1-weighted images (T1WI), the lesion was mainly hypointense, but with some nodular hyperintensity **(D)**. Contrast-enhanced images showed the markedly heterogeneous enhancement of the tumor **(E, F)**, and tissue with hypointensity on T2WI could be significantly enhanced or not (*white* and *black arrowheads*). The tumor demonstrated internal low-signal septations on sagittal T2-weighted fat-suppressed acquisitions resembling the pattern of a human brain (“pseudo-cerebriform” appearance) **(G)**.

**Figure 4:**
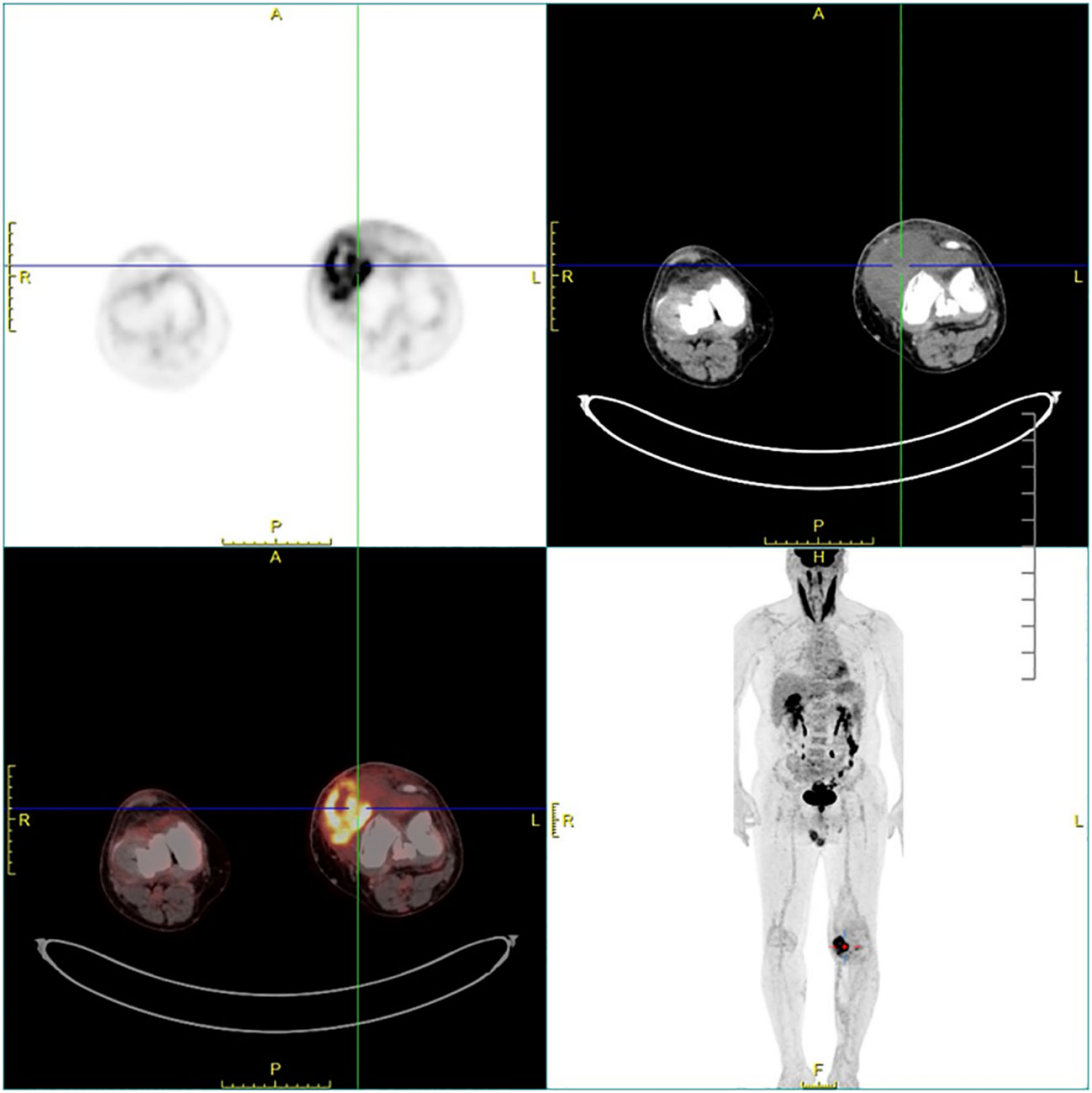
Fluorodeoxyglucose F18 (F-18 FDG) PET-CT showing an active heterogeneously enhanced mass in the joint cavity of the left knee.

Ethics approval for this study was granted by the Institutional Review Board of our hospital. A 60-year-old man was admitted to the Department of Orthopedics of our hospital due to swelling in the left knee for the past 8 months. The patient had no history of relevant trauma and had no other complaints of discomfort. The skin and the temperature of the left knee were normal. X-ray of the left knee was first taken (**Figure 1**), which showed soft tissue swelling mainly on the anteromedial side of the knee, where the density was higher than that of normal soft tissue. Moreover, some strip calcification could be seen in the X-ray, but there was no destruction of the bone. To further define the lesion, the radiologist recommended CT or MRI. Enhanced CT and MRI were both performed, which confirmed the irregular soft tissue mass within the joint cavity of the left knee. CT of the left knee (**Figure 2**) showed that the calcification in the mass was mainly located at the edge. After injection of a contrast material, the enhancement was heterogeneous, and a nodular enhancement of the lesion was presented. Small enhanced vessels could be seen in and around the mass. There was no destruction to the adjacent bones. MRI presented the shape of the lesion better (**Figure 3**). The mass was irregular, and it appeared to grow along the synovial membrane of the joint and was partly nodular. On T2-weighted images (T2WI), the lesion was hyperintense. On T1-weighted images (T1WI), the lesion was mainly hypointense, but with some nodular hyperintensity. In the enhanced images, the lesion showed a markedly heterogeneous enhancement, especially the nodules. The margin of the lesion was unclear. The adjacent medial collateral ligament of the left knee was swollen and was partly invaded by the mass. Edema was evident in the soft tissue surrounding the mass. It was difficult to determine what the lesion was; therefore, a needle biopsy was performed, which suggested malignancy. F-18 FDG PET-CT, which was performed for tumor restaging, revealed active heterogeneous enhancement of only one tumor, with a maximum standardized uptake value (SUV_max_) of 7.51 (**Figure 4**). No other metabolically active lesions were seen in the body; therefore, the patient underwent surgical resection. The soft tissue and muscle around the tumor were separated, and the distal femur and the proximal tibia and fibula were excised for complete resection of the tumor. Subsequently, knee prosthesis replacement was performed. The whole operation was successful. Postoperative pathology confirmed malignant SFT with focal necrosis. Immunohistochemical examination revealed that the neoplastic cells were positive for CD34, Bcl-2, and SATA6 (**Supplementary Figure S1**). The immunohistochemistry results were as follows: CD34 (partially ++), Bcl-2 (partially ++), STAT6 (weak +), EMA (focal +), desmin (−), S-100 (−), beta-catenin (focal +), SMA (focal +), CK(AE1/AE3) (scattered +), CAM5.2 (scattered +), and Ki-67 (30% +). Approximately 11 months after surgery, the patient came to our hospital again due to bone abnormalities, and it was confirmed by imaging examination and puncture pathology that the patient had multiple bone metastases (**Supplementary Figure S2**).

## Discussion

SFTs are rare tumors accounting for less than 2% of soft tissue tumors (12, 13). SFTs most commonly occur in the pleura, but approximately 10%–25% can derive from soft tissues, most commonly in the proximal lower limbs, usually deep. In terms of the imaging features of SFTs, they commonly present as large, well-circumscribed, hypervascular masses deep to fascia along major neurovascular bundles (14). In this report, we described a rare case of a malignant SFT within the joint cavity of the left knee.

At present, imaging findings of SFTs in soft tissue are mainly presented as individual or small case series reports (14–30). To our knowledge, the largest case series published to date is that by Swami et al., which showed the MRI features of 39 patients (14). The existing literature on the imaging features of SFTs in soft tissue usually describe them as located in the lower extremity and often deep to fascia along major neurovascular bundles. A small proportion of SFTs can occur in superficial sites, such as the abdominal wall. The case reported here occurred in the left lower limb, which was consistent with previous reports; however, its growth was mainly located in the cavity of the knee joint. Garcia-Bennett et al. previously reported on the case of a 77-year-old female patient with SFT (29) whose tumor affected the intra- and extra-articular fat with disruption of the medial patellar retinaculum of the knee. However, unlike that in our case, the tumor mainly grew in the intra-articular space, which is extremely rare and, to our knowledge, has not been previously reported. Most SFTs presented a well-defined lobulated mass in previous studies (14). In our case, however, the tumor did not have a clump-like shape. It was ill-defined and appeared more like a mass growing along the intraluminal synovium, which may be an indication of its malignancy. The rare site of occurrence and the morphology of the mass led to this being a rare case. On MRI, there were various results of T1 and T2 signal presentation of SFT in soft tissue reported. On T2WI, most studies described SFTs as heterogeneous, but predominantly hyperintense (26, 27), while some were described as heterogeneous with mixed hyper- and hypointense signals (28, 29). On T1WI, a number of studies described SFTs as isointense to muscle (26–28), while others were described as isointense-to-hypointense (23) or isointense-to-hyperintense (29). The diversity of the MRI signal features on T1- and T2-weighted imaging could be due to the influence of the tumor cellular variant composition, which was difficult to characterize in previous studies. In our case, the tumor was heterogeneous, with mixed hypointense and hyperintense signals on both T1- and T2-weighted imaging. In the fluid-sensitive sequences, a feature resembling morphologic internal lobulations with intra-lesional low-T2 signal bands/septations on a background of heterogenous signal was demonstrated (**Figure 3**), which we believed might be the “human brain” or the “pseudo-cerebriform” sign on the T2/STIR-weighted fat-suppressed MRI acquisitions, as Swami et al. reported (14).

Soft tissue SFTs are classically described as hypervascular lesions; however, a number of studies found a feature of peripheral extracapsular vessels forming an apparent vascular pedicle, which was thought to more likely occur in larger lesions. This was observed not only in SFTs of soft tissue (14, 26, 29), but also of the pleura and retroperitoneum (31–33). Some studies recommended the feature of large collateral feeding vessels at a vascular pedicle as a possible distinguishing feature of SFTs compared with other hypervascular masses (27, 29), but its diagnostic specificity has not been carefully assessed. In our case, the peripheral and intra-lesional vessels were enhanced on CT imaging, and there were many large collateral feeding vessels around the mass, which was similar to previous studies to a certain extent. The enhancement pattern of soft tissue SFTs is often heterogeneous (14, 26–29, 34, 35), reflecting the high degree of vascularity. Some internal areas without enhancement, after injection of a contrast medium, corresponded with high signals on T1 and T2 images. We believed they might represent areas of tumor necrosis in histology.

Calcification is rare in SFTs. In our case, the calcification was patchy and located at the edge of the mass, which made it more difficult to distinguish from synovial sarcoma, which typically presents as a mass around large joints with obvious mixed signals and eccentric calcification occurring in young adults. Different forms of calcification in SFTs have been reported previously, including tiny, scattered, partial, or prominent calcification (27, 36, 37). Some researchers believe that calcification could be regarded as an indicator of malignancy (38), but its reliability has not been proven. In our case, the F-18 FDG PET-CT showed good uptake in the primary tumor and no other lesions were detected, which helped in the staging. There are only a few descriptions in the literature with regard to the PET manifestations of SFTs. It was found that benign SFTs exhibit low-grade activity in PET and that malignant SFTs tend to be strongly hypermetabolic and homogeneous (10).

The standard treatment for SFTs is still radical resection with long-term follow-up due to 5%–10% of patients experiencing recurrence or metastasis. The fifth edition of WHO recommended the use of a risk stratification model modified by Demicco et al. (39), which includes age (<55 years or ≥55 years), tumor size (<5, 5–10, 10–15 cm, or ≥15 cm), mitotic count (0, 1–3, or ≥4 per 10 high-magnification fields), and tumor necrosis (<10% or ≥10%). The scores are then added together to create a risk rating: 0–3 points for low risk, 4–5 points for moderate risk, and 6–7 points for high risk. According to this criterion, our patient had a total score of 7, indicating a high risk of metastasis. Adjuvant radiotherapy can be considered for high-risk, inoperable, or locally recurrent lesion, but its survival benefit is still under investigation. The patient in this study did not receive radiotherapy after surgery. Unfortunately, 11 months after surgery, the patient developed bone metastases. Ultimately, benign SFTs have a local recurrence rate of 8%, and malignant lesions recur within 2 years in as many as 63% of cases (40).

## Conclusions

We reported on a relatively uncommon case of malignant SFT within the joint cavity of the left knee, which presented as a large, ill-circumscribed, hypervascular mass with eccentric calcification and was diagnosed by immunohistochemical examination as positive for CD34, Bcl-2, and SATA6. The patient developed bone metastases within 1 year after surgery. This is the first case of SFT in the joint cavity, and radiologists should be aware of the possibility of this rare origin. Soft tissue SFTs are difficult to diagnose and should be carefully followed up after surgery.

## Data Availability

The original contributions presented in the study are included in the article/**Supplementary Material**. Further inquiries can be directed to the corresponding author.

## Supplementary material

Supplementary Figure 1 Histology of resected tumor. Immunohistochemistry results were as follows: CD34 (partially ++), Bcl-2 (partially ++), STAT6 (weak +), EMA (focal +), Desmin (-), S-100 (-), Beta-catenin (focal +), SMA (focal +), CK(AE1/AE3) (Scattered +), CAM5.2 (Scattered +), Ki-67 (30% +).

Supplementary Figure 2 MRI of the Lumbosacral vertebrae was obtained at about 11 months after surgery. **(A)** Sagittal l T1 weighted MRI shows hypointense in multiple lumbosacral vertebrae, replacing the normal medullary bone. **(B)** Axial T2-weighted fat-suppressed image shows that these lesions were heterogeneously little high signal intensity. (C, D) Coronal T1-weighted fat-suppressed image gadolinium-enhanced image shows these lesions were significantly uneven enhancing, and Necrotic areas were found in some lesions. The same findings were found in the iliac crest, the pubic bone, and the right proximal femur. The patient underwent a needle biopsy and the pathology was consistent with SFT.

## References

[1] Klemperer P Coleman BR. Primary neoplasms of the pleura. A report of five cases. Am J Ind Med. (1992) 22:1–31. doi: 10.1002/ajim.4700220103 1415270

[2] Sbaraglia M Bellan E Dei Tos AP. The 2020 WHO Classification of Soft Tissue Tumours: news and perspectives. Pathologica. (2021) 113:70–84. doi: 10.32074/1591-951X-213 33179614 PMC8167394

[3] Gengler C Guillou L. Solitary fibrous tumour and haemangiopericytoma: evolution of a concept. Histopathology. (2006) 48:63–74. doi: 10.1111/j.1365-2559.2005.02290.x 16359538

[4] Verbeke SL Fletcher CD Alberghini M Daugaard S Flanagan AM Parratt T. A reappraisal of hemangiopericytoma of bone; analysis of cases reclassified as synovial sarcoma and solitary fibrous tumor of bone. Am J Surg Pathol. (2010) 34:777–83. doi: 10.1097/PAS.0b013e3181dbedf1 20421780

[5] Doyle LA Vivero M Fletcher CD Mertens F Hornick JL. Nuclear expression of STAT6 distinguishes solitary fibrous tumor from histologic mimics. Mod Pathol. (2014) 27:390–5. doi: 10.1038/modpathol.2013.164 24030747

[6] Jia C Crim J Evenski A Layfield LJ. Solitary fibrous tumor of bone developing lung metastases on long-term follow-up. Skeletal Radiol. (2020) 49:1865–71. doi: 10.1007/s00256-020-03493-x 32519181

[7] Brunnemann RB Ro JY Ordonez NG Mooney J El-Naggar AK Ayala AG. Extrapleural solitary fibrous tumor: a clinicopathologic study of 24 cases. Mod Pathol. (1999) 12:1034–42. 10574600

[8] Fukunaga M Naganuma H Nikaido T Harada T Ushigome S. Extrapleural solitary fibrous tumor: a report of seven cases. Mod Pathol. (1997) 10:443–50. 9160308

[9] Son S Lee SG Jeong DH Yoo CJ. Malignant solitary fibrous tumor of tandem lesions in the skull and spine. J Korean Neurosurg Soc. (2013) 54:246–9. doi: 10.3340/jkns.2013.54.3.246 PMC3836935 24278657

[10] Ginat DT Bokhari A Bhatt S Dogra V. Imaging features of solitary fibrous tumors. AJR Am J Roentgenol. (2011) 196:487–95. doi: 10.2214/AJR.10.4948 21343490

[11] Prakash S Shamim SA Rastogi S Barwad A. A rare case of solitary fibrous tumor of maxilla: findings on F-18 FDG and ga-68 DOTANOC PET-CT. Nucl Med Mol Imaging. (2023) 57:34–7. doi: 10.1007/s13139-022-00768-0 PMC9832199 36643945

[12] Suarez-Zamora DA Rodriguez-Urrego PA Soto-Montoya C Rivero-Rapalino O Palau-Lazaro MA. Malignant solitary fibrous tumor of the humerus: A case report of an extremely rare primary bone tumor. Int J Surg Pathol. (2018) 26:772–6. doi: 10.1177/1066896918780348 29961401

[13] Kayani B Sharma A Sewell MD Platinum J Olivier A Briggs TWR. A review of the surgical management of extrathoracic solitary fibrous tumors. Am J Clin Oncol. (2018) 41:687–94. doi: 10.1097/COC.0000000000000348 27893469

[14] Swami VG Demicco EG Naraghi A White LM. Soft tissue solitary fibrous tumors of the musculoskeletal system: spectrum of MRI appearances and characteristic imaging features. Skeletal Radiol. (2022) 51:807–17. doi: 10.1007/s00256-021-03894-6 34430995

[15] Abe S Imamura T Tateishi A Park P Nakano H Harasawa A. Intramuscular solitary fibrous tumor: a clinicopathological case study. J Comput Assist Tomogr. (1999) 23:458–62. doi: 10.1097/00004728-199905000-00024 10348456

[16] Anders JO Aurich M Lang T Wagner A. Solitary fibrous tumor in the thigh: review of the literature. J Cancer Res Clin Oncol. (2006) 132:69–75. doi: 10.1007/s00432-005-0055-7 16283380 PMC12161049

[17] Martorell M Pérez-Vallés A Gozalbo F Garcia-Garcia JA Gutierrez J Gaona J. Solitary fibrous tumor of the thigh with epithelioid features: a case report. Diagn Pathol. (2007) 2:19. doi: 10.1186/1746-1596-2-19 17577399 PMC1913496

[18] Rakheja D Wilson KS Meehan JJ Schultz RA Maale GE Timmons CF. Extrapleural benign solitary fibrous tumor in the shoulder of a 9-year-old girl: case report and review of the literature. Pediatr Dev Pathol. (2004) 7:653–60. doi: 10.1007/s10024-004-6065-7 15630539

[19] Kean CA Moore BR Nettles AM Bui RP. Extrapleural solitary fibrous tumor of the foot A case report. J Am Podiatr Med Assoc. (2015) 105:557–9. doi: 10.7547/13-126.1 26667509

[20] Lee JY Kim DH Seo KJ Jung SN. A solitary fibrous tumor (Cellular form) of the ankle. J Foot Ankle Surg. (2016) 55:829–31. doi: 10.1053/j.jfas.2015.03.006 25979291

[21] Al-Shanawani BN Al-Qattan MM Arafah MM Al-Motairi MI. A solitary fibrous tumor of the upper limb. Saudi Med J. (2015) 36:236–8. doi: 10.15537/smj.2015.2.10524 PMC4375705 25719592

[22] Yamamoto Y Kanzaki R Inoue M Okumura M. Primary solitary fibrous tumor of the forearm with frequent late-onset recurrence in the pleura. Ann Thorac Surg. (2017) 104:e173–5. doi: 10.1016/j.athoracsur.2017.03.036 28734445

[23] Insabato L Siano M Somma A Gentile R Santangelo M Pettinato G. Extrapleural solitary fibrous tumor: a clinicopathologic study of 19 cases. Int J Surg Pathol. (2009) 17:250–4. doi: 10.1177/1066896909333779 19443888

[24] Daigeler A Lehnhardt M Langer S Steinstraesser L Steinau HU Mentzel T. Clinicopathological findings in a case series of extrathoracic solitary fibrous tumors of soft tissues. BMC Surg. (2006) 6:10. doi: 10.1186/1471-2482-6-10 16824225 PMC1523192

[25] Akisue T Matsumoto K Kizaki T Fujita I Yamamoto T Yoshiya S. Solitary fibrous tumor in the extremity: case report and review of the literature. Clin Orthop Relat Res. (2003) 411:236–44. doi: 10.1097/01.blo.0000065839.77325.b4 12782880

[26] Hyodo R Komada T Takada A Kawai H Ito S Nishida Y. Solitary fibrous tumors in the extremities: imaging findings for six patients. Nagoya J Med Sci. (2015) 77:167–78. PMC4361518 25797981

[27] Wignall OJ Moskovic EC Thway K Thomas JM. Solitary fibrous tumors of the soft tissues: review of the imaging and clinical features with histopathologic correlation. AJR Am J Roentgenol. (2010) 195:W55–62. doi: 10.2214/AJR.09.3379 20566782

[28] Papathanassiou ZG Alberghini M Picci P Staals E Gambarotti M Garaci FG. Solitary fibrous tumors of the soft tissues: imaging features with histopathologic correlations. Clin Sarcoma Res. (2013) 3:1. doi: 10.1186/2045-3329-3-1 23351922 PMC3637805

[29] Garcia-Bennett J Olivé CS Rivas A Domínguez-Oronoz R Huguet P. Soft tissue solitary fibrous tumor. Imaging findings in a series of nine cases. Skeletal Radiol. (2012) 41:1427–33. doi: 10.1007/s00256-012-1364-y 22349595

[30] Thway K Ng W Noujaim J Jones RL Fisher C. The current status of solitary fibrous tumor: diagnostic features, variants, and genetics. Int J Surg Pathol. (2016) 24:281–92. doi: 10.1177/1066896915627485 26811389

[31] de Perrot M Fischer S Bründler MA Sekine Y Keshavjee S. Solitary fibrous tumors of the pleura. Ann Thorac Surg. (2002) 74:285–93. doi: 10.1016/S0003-4975(01)03374-4 12118790

[32] Tateishi U Nishihara H Morikawa T Miyasaka K. Solitary fibrous tumor of the pleura: MR appearance and enhancement pattern. J Comput Assist Tomogr. (2002) 26:174–9. doi: 10.1097/00004728-200203000-00002 11884769

[33] Wat SY Sur M Dhamanaskar K. Solitary fibrous tumor (SFT) of the pelvis. Clin Imaging. (2008) 32:152–6. doi: 10.1016/j.clinimag.2007.07.003 18313582

[34] Keraliya AR Tirumani SH Shinagare AB Zaheer A Ramaiya NH. Solitary fibrous tumors: 2016 imaging update. Radiol Clin North Am. (2016) 54:565–79. doi: 10.1016/j.rcl.2015.12.006 27153789

[35] Musyoki FN Nahal A Powell TI. Solitary fibrous tumor: an update on the spectrum of extrapleural manifestations. Skeletal Radiol. (2012) 41:5–13. doi: 10.1007/s00256-010-1032-z 20953607

[36] Maki T Fujino S Misu K Kaneko H Inomata H Omi M. Integrally calcified solitary fibrous tumor in the retroperitoneum: a case report and review of the literature. Surg Case Rep. (2016) 2:14. doi: 10.1186/s40792-016-0143-8 26943690 PMC4752942

[37] Rekhi B Bapat P Chakrabarty N Nayak P. A case of a large solitary fibrous tumor in the thigh, displaying NAB2ex4-STAT6ex2 gene fusion. Skeletal Radiol. (2021) 50:2299–307. doi: 10.1007/s00256-021-03829-1 34052867

[38] Li JP Xie CM Zhang R Li H Liu XW Zhang Y. Imaging features and clinicopathological manifestations of solitary fibrous tumors. Zhonghua Zhong Liu Za Zhi. (2010) 32:363–7. 20723435

[39] Demicco EG Wagner MJ Maki RG Gupta V Iofin I Lazar AJ. Risk assessment in solitary fibrous tumors: validation and refinement of a risk stratification model. Mod Pathol. (2017) 30:1433–42. doi: 10.1038/modpathol.2017.54 28731041

[40] Robinson LA. Solitary fibrous tumor of the pleura. Cancer Control. (2006) 13:264–9. doi: 10.1177/107327480601300403 17075563

