# Supplementary figures and images for: Case report: A rare case of malignant solitary fibrous tumor within the joint cavity with review of the literature
Source: Front Oncol. 2024 Nov 29;14:1463362. doi: 10.3389/fonc.2024.1463362 (PMC11638052; doi:10.3389/fonc.2024.1463362)

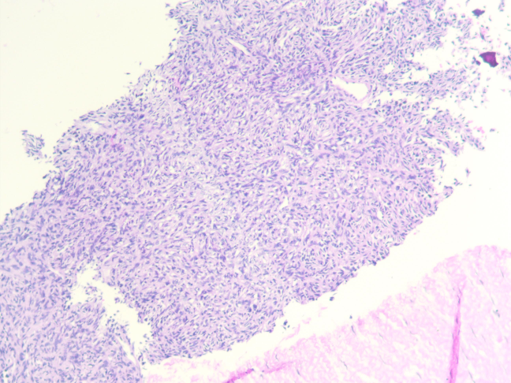

Supplement: Supplementary Figure 1 — Histology of resected tumor. Immunohistochemistry results were as follows: CD34 (partially ++), Bcl-2 (partially ++), STAT6 (weak +), EMA (focal +), Desmin (-), S-100 (-), Beta-catenin (focal +), SMA (focal +), CK(AE1/AE3) (Scattered +), CAM5.2 (Scattered +), Ki-67 (30% +). [file Image1.tif]

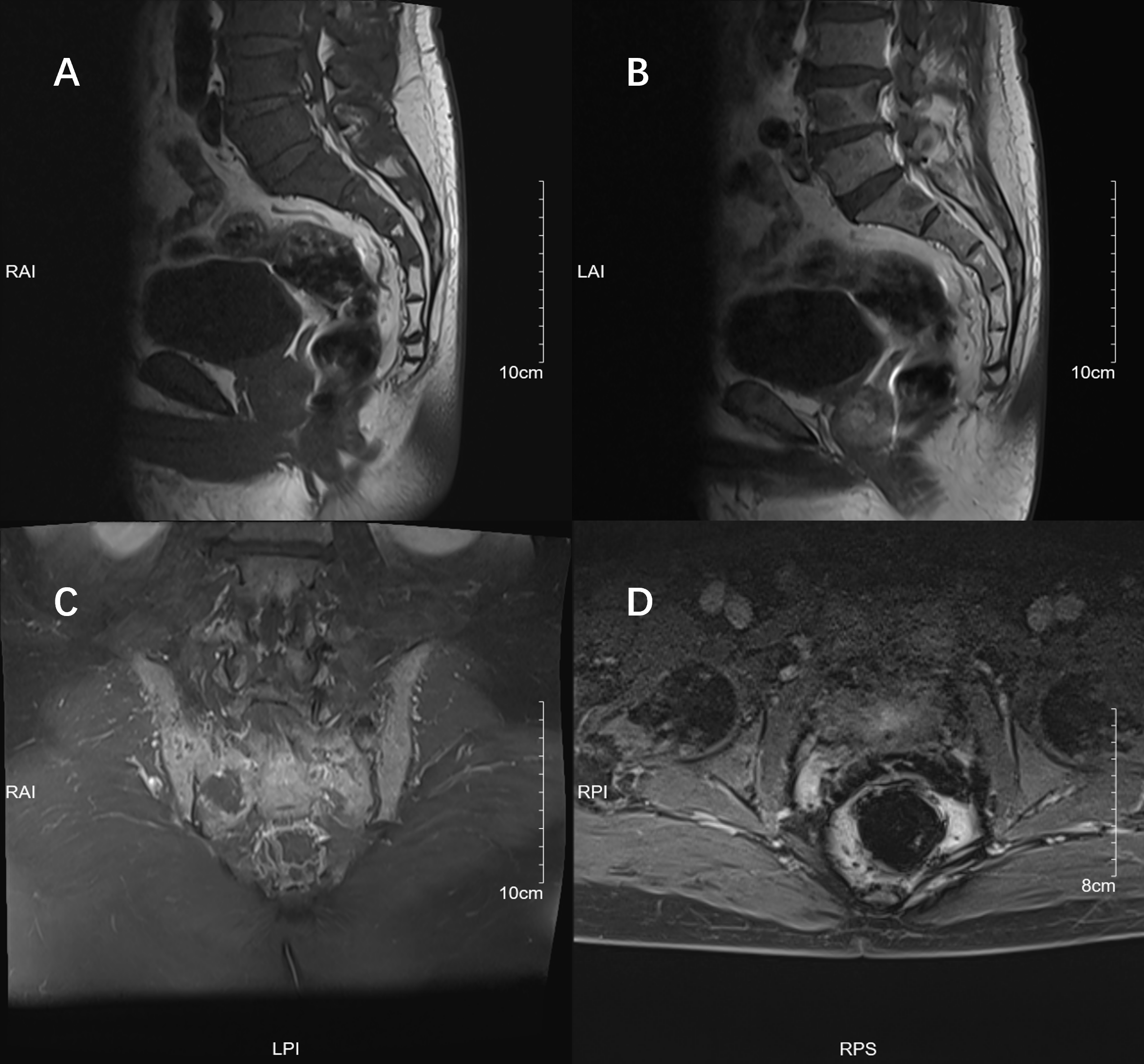

Supplement: Supplementary Figure 2 — MRI of the Lumbosacral vertebrae was obtained at about 11 months after surgery. (A) Sagittal l T1 weighted MRI shows hypointense in multiple lumbosacral vertebrae, replacing the normal medullary bone. (B) Axial T2-weighted fat-suppressed image shows that these lesions were heterogeneously little high signal intensity. (C, D) Coronal T1-weighted fat-suppressed image gadolinium-enhanced image shows these lesions were significantly uneven enhancing, and Necrotic areas were found in some lesions. The same findings were found in the iliac crest, the pubic bone, and the right proximal femur. The patient underwent a needle biopsy and the pathology was consistent with SFT. [file Image2.tif]
